# Supplementary material for: Cloud BioLinux: pre-configured and on-demand bioinformatics computing for the genomics community
Source: BMC Bioinformatics. 2012 Mar 19;13:42. doi: 10.1186/1471-2105-13-42 (PMC3372431; doi:10.1186/1471-2105-13-42)
Supplement: Additional file 1 — Supplementary 1 Cloud BioLinux software documentation in the form of a mini, self-contained website. Users need to download and uncompress the .zip file, and open through a web browser the "index.html" file available on the main directory. (ZIP 1823 kb). [file 1471-2105-13-42-S1.ZIP › Cloud-BioLinux-Package-Documentation/Feature tables.html]

Bio-Linux Software Documentation Pages

Back to search form

### Feature tables

|  |  |
| --- | --- |
| Category | Applications |
| Feature tables | coderet    coderet    coderet    coderet    extractfeat    extractfeat    extractfeat    extractfeat    maskfeat    maskfeat    maskfeat    maskfeat    showfeat    showfeat    showfeat    showfeat    twofeat    twofeat    coderet    coderet    coderet    coderet    extractfeat    extractfeat    extractfeat    extractfeat    maskfeat    maskfeat    maskfeat    maskfeat    showfeat    showfeat    showfeat    showfeat    twofeat    twofeat    coderet    coderet    coderet    coderet    extractfeat    extractfeat    extractfeat    extractfeat    maskfeat    maskfeat    maskfeat    maskfeat    showfeat    showfeat    showfeat    showfeat    twofeat    twofeat |
| SubCategory | Applications |
|  | abiview    abiview    abiview    abiview    abiview    abiview    acdc    acdc    acdc    acdc    acdc    acdc    acdlog    acdlog    acdlog    acdlog    acdlog    acdlog    acdpretty    acdpretty    acdpretty    acdpretty    acdpretty    acdpretty    acdtable    acdtable    acdtable    acdtable    acdtable    acdtable    acdtrace    acdtrace    acdtrace    acdtrace    acdtrace    acdtrace    acdvalid    acdvalid    acdvalid    acdvalid    acdvalid    acdvalid    ajbad    ajbad    ajbad    ajbad    ajbad    ajbad    ajfeatest    ajfeatest    ajfeatest    ajfeatest    ajfeatest    ajfeatest    ajtest    ajtest    ajtest    ajtest    ajtest    ajtest    aligncopy    aligncopy    aligncopy    aligncopy    aligncopy    aligncopy    aligncopypai    aligncopypai    aligncopypai    aligncopypai    aligncopypai    aligncopypai    biosed    biosed    biosed    biosed    biosed    biosed    cirdna    cirdna    cirdna    cirdna    cirdna    cirdna    codcopy    codcopy    codcopy    codcopy    codcopy    codcopy    coderet    coderet    coderet    coderet    coderet    coderet    coderet    coderet    coderet    coderet    coderet    coderet    corbatest    corbatest    corbatest    corbatest    corbatest    corbatest    cutseq    cutseq    cutseq    cutseq    cutseq    cutseq    degapseq    degapseq    degapseq    degapseq    degapseq    degapseq    demoalign    demoalign    demoalign    demoalign    demoalign    demoalign    demofeatures    demofeatures    demofeatures    demofeatures    demofeatures    demofeatures    demolist    demolist    demolist    demolist    demolist    demolist    demoreport    demoreport    demoreport    demoreport    demoreport    demoreport    demosequence    demosequence    demosequence    demosequence    demosequence    demosequence    demostring    demostring    demostring    demostring    demostring    demostring    demostringnew    demostringnew    demostringnew    demostringnew    demostringnew    demostringnew    demotable    demotable    demotable    demotable    demotable    demotable    descseq    descseq    descseq    descseq    descseq    descseq    entret    entret    entret    entret    entret    entret    extractalign    extractalign    extractalign    extractalign    extractalign    extractalign    extractfeat    extractfeat    extractfeat    extractfeat    extractfeat    extractfeat    extractfeat    extractfeat    extractfeat    extractfeat    extractfeat    extractfeat    extractfeat    extractfeat    extractfeat    extractfeat    extractfeat    extractfeat    extractfeat    extractfeat    extractfeat    extractfeat    extractfeat    extractfeat    extractseq    extractseq    extractseq    extractseq    extractseq    extractseq    featcopy    featcopy    featcopy    featcopy    featcopy    featcopy    featreport    featreport    featreport    featreport    featreport    featreport    findkm    findkm    findkm    findkm    findkm    findkm    histogramtest    histogramtest    histogramtest    histogramtest    histogramtest    histogramtest    infoalign    infoalign    infoalign    infoalign    infoalign    infoalign    infoalign    infoalign    infoalign    infoalign    infoalign    infoalign    infobas    infobas    infobas    infobas    infobas    infobas    inforesidu    inforesidu    inforesidu    inforesidu    inforesidu    inforesidu    infoseq    infoseq    infoseq    infoseq    infoseq    infoseq    intconv    intconv    intconv    intconv    intconv    intconv    lindna    lindna    lindna    lindna    lindna    lindna    listor    listor    listor    listor    listor    listor    makenucseq    makenucseq    makenucseq    makenucseq    makenucseq    makenucseq    makeprotseq    makeprotseq    makeprotseq    makeprotseq    makeprotseq    makeprotseq    maskambignuc    maskambignuc    maskambignuc    maskambignuc    maskambignuc    maskambignuc    maskambigprot    maskambigprot    maskambigprot    maskambigprot    maskambigprot    maskambigprot    maskfeat    maskfeat    maskfeat    maskfeat    maskfeat    maskfeat    maskfeat    maskfeat    maskfeat    maskfeat    maskfeat    maskfeat    maskfeat    maskfeat    maskfeat    maskfeat    maskfeat    maskfeat    maskfeat    maskfeat    maskfeat    maskfeat    maskfeat    maskfeat    maskseq    maskseq    maskseq    maskseq    maskseq    maskseq    newseq    newseq    newseq    newseq    newseq    newseq    nohtm    nohtm    nohtm    nohtm    nohtm    nohtm    noreturn    noreturn    noreturn    noreturn    noreturn    noreturn    nospac    nospac    nospac    nospac    nospac    nospac    nota    nota    nota    nota    nota    nota    notseq    notseq    notseq    notseq    notseq    notseq    nthseq    nthseq    nthseq    nthseq    nthseq    nthseq    origsplitter    origsplitter    origsplitter    origsplitter    origsplitter    origsplitter    origunion    origunion    origunion    origunion    origunion    origunion    pasteseq    pasteseq    pasteseq    pasteseq    pasteseq    pasteseq    patmattest    patmattest    patmattest    patmattest    patmattest    patmattest    pepnet    pepnet    pepnet    pepnet    pepnet    pepnet    pepnet    pepnet    pepnet    pepnet    pepnet    pepnet    pepwheel    pepwheel    pepwheel    pepwheel    pepwheel    pepwheel    pepwheel    pepwheel    pepwheel    pepwheel    pepwheel    pepwheel    prettyplot    prettyplot    prettyplot    prettyplot    prettyplot    prettyplot    prettyplot    prettyplot    prettyplot    prettyplot    prettyplot    prettyplot    prettyseq    prettyseq    prettyseq    prettyseq    prettyseq    prettyseq    prettyseq    prettyseq    prettyseq    prettyseq    prettyseq    prettyseq    remap    remap    remap    remap    remap    remap    remap    remap    remap    remap    remap    remap    remap    remap    remap    remap    remap    remap    revseq    revseq    revseq    revseq    revseq    revseq    seealso    seealso    seealso    seealso    seealso    seealso    seealso    seealso    seealso    seealso    seealso    seealso    seealso    seealso    seealso    seealso    seealso    seealso    seealso    seealso    seealso    seealso    seealso    seealso    seqinfo    seqinfo    seqinfo    seqinfo    seqinfo    seqinfo    seqret    seqret    seqret    seqret    seqret    seqret    seqretall    seqretall    seqretall    seqretall    seqretall    seqretall    seqretallfeat    seqretallfeat    seqretallfeat    seqretallfeat    seqretallfeat    seqretallfeat    seqretset    seqretset    seqretset    seqretset    seqretset    seqretset    seqretsingle    seqretsingle    seqretsingle    seqretsingle    seqretsingle    seqretsingle    seqretsplit    seqretsplit    seqretsplit    seqretsplit    seqretsplit    seqretsplit    seqrettype    seqrettype    seqrettype    seqrettype    seqrettype    seqrettype    showalign    showalign    showalign    showalign    showalign    showalign    showalign    showalign    showalign    showalign    showalign    showalign    showdb    showdb    showdb    showdb    showdb    showdb    showdb    showdb    showdb    showdb    showdb    showdb    showdb    showdb    showdb    showdb    showdb    showdb    showdb    showdb    showdb    showdb    showdb    showdb    showfeat    showfeat    showfeat    showfeat    showfeat    showfeat    showfeat    showfeat    showfeat    showfeat    showfeat    showfeat    showfeat    showfeat    showfeat    showfeat    showfeat    showfeat    showfeat    showfeat    showfeat    showfeat    showfeat    showfeat    showseq    showseq    showseq    showseq    showseq    showseq    showseq    showseq    showseq    showseq    showseq    showseq    showseq    showseq    showseq    showseq    showseq    showseq    sixpack    sixpack    sixpack    sixpack    sixpack    sixpack    sixpack    sixpack    sixpack    sixpack    sixpack    sixpack    sixpack    sixpack    sixpack    sixpack    sixpack    sixpack    sizese    sizese    sizese    sizese    sizese    sizese    skipredundan    skipredundan    skipredundan    skipredundan    skipredundan    skipredundan    skipseq    skipseq    skipseq    skipseq    skipseq    skipseq    splitter    splitter    splitter    splitter    splitter    splitter    testplot    testplot    testplot    testplot    testplot    testplot    textsearch    textsearch    textsearch    textsearch    textsearch    textsearch    textsearch    textsearch    textsearch    textsearch    textsearch    textsearch    textsearch    textsearch    textsearch    textsearch    textsearch    textsearch    textsearch    textsearch    textsearch    textsearch    textsearch    textsearch    tfm    tfm    tfm    tfm    tfm    tfm    treetypedisplay    treetypedisplay    treetypedisplay    treetypedisplay    treetypedisplay    treetypedisplay    trimest    trimest    trimest    trimest    trimest    trimest    trimseq    trimseq    trimseq    trimseq    trimseq    trimseq    trimspac    trimspac    trimspac    trimspac    trimspac    trimspac    twofeat    twofeat    twofeat    twofeat    twofeat    twofeat    union    union    union    union    union    union    vectorstrip    vectorstrip    vectorstrip    vectorstrip    vectorstrip    vectorstrip    whichdb    whichdb    whichdb    whichdb    whichdb    whichdb    wossname    wossname    wossname    wossname    wossname    wossname    yank    yank    yank    yank    yank    yank |
